# Quantitative EEG Markers of Elevated Intracranial Pressure in a Case of Carcinomatous Meningitis

**DOI:** 10.1007/s12028-025-02372-4

**Published:** 2025-10-13

**Authors:** Cody L. Nathan, Diamond A. Dominguez, Elizabeth Gerard

**Affiliations:** Northwestern Memorial Hospital, Chicago, IL USA

## Abstract

We present the case of a 69-year-old woman with acute myeloid leukemia with recurrent transient events characterized by loss of awareness and generalized shaking. The events were initially diagnosed as seizures based on semiology but persisted despite antiseizure medications. The events were recorded using continuous video electroencephalography (EEG) with no epileptiform correlate. However, pertinent changes included diffuse attenuation of fast activity, increase in delta activity, and subsequent attenuation of faster frequencies. Quantitative EEG detected a decrease in fast activity, alpha-delta ratio, and amplitude-integrated EEG. The transient events and EEG findings in the setting of known cancer history raised concern for carcinomatous meningitis despite unremarkable brain imaging. Lumbar puncture showed an elevated opening pressure and cytology confirmed a myeloid blast population consistent with acute myeloid leukemia. The events resolved with serial lumbar punctures supporting the fact that the events were likely secondary to transient elevations in intracerebral pressure. In summary, video EEG with quantitative EEG analysis is a sensitive, non-invasive way to confirm transient elevated intracranial pressure and rule out epileptic activity. This constellation of cancer, clinical symptoms and EEG findings should increase suspicion of carcinomatous meningitis.

## Case Description

**Fig. 1:**
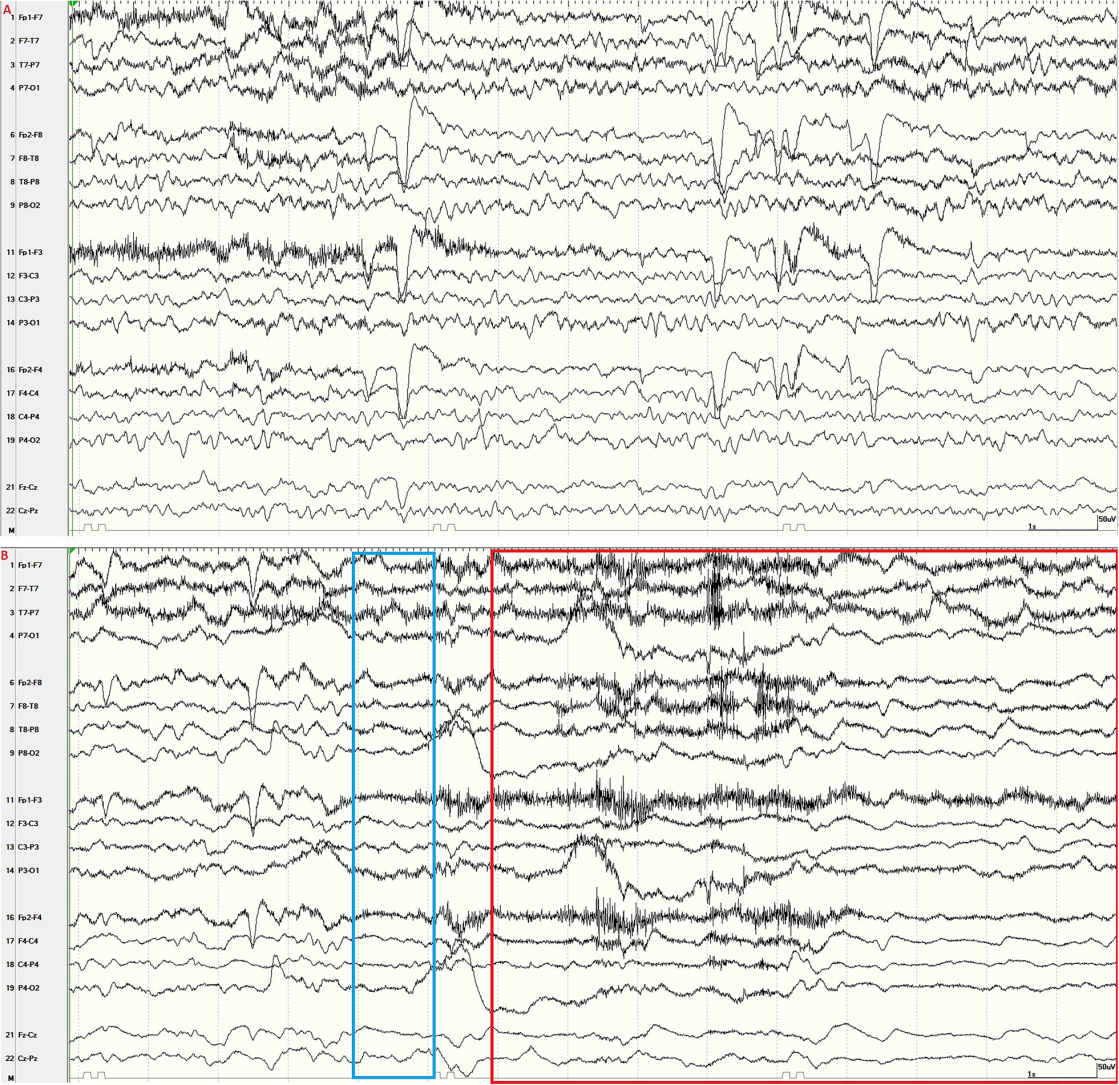
Baseline EEG compared with EEG at the onset of an event; 10–20 EEG, longitudinal bipolar montage, 15 mm/s, sensitivity 7 µV/mm. **a** Baseline EEG noting a symmetric background with a normal posterior dominant rhythm. There is continuous generalized slowing but no epileptiform abnormalities. **b** Onset of the clinical event correlates with an abrupt attenuation of alpha activity (red box) and emergence of diffuse, low-amplitude delta activity (blue box) with superimposed electromyography artifact. This attenuation of fast activity is a profound change compared with the patient’s baseline EEG as noted in **a**. EEG, electroencephalogram

**Fig. 2:**
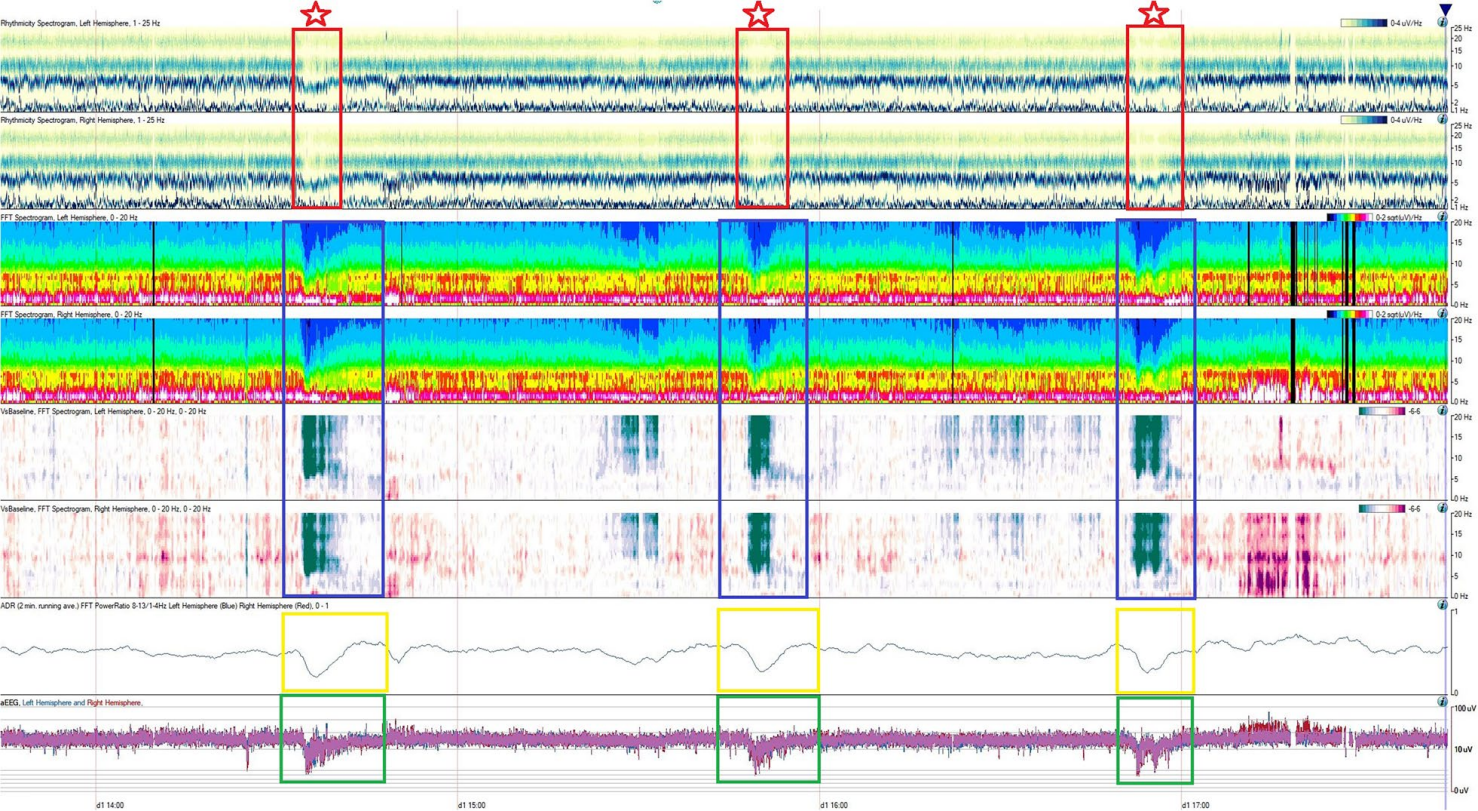
Quantitative EEG findings during three events. Quantitative EEG over four hours (time denoted in the *x*-axis), with each row indicating a different quantitative EEG measure. There are three clinical events (demarcated by red stars). At the onset of the events, there is a sudden decrease in rhythmicity at higher frequencies (red boxes). Fast Fourier transform (blue boxes) is a mathematical technique that shows EEG power contained within certain frequency bands. With each event, there is a sudden loss of power for faster frequencies across bilateral hemispheres. There is also an associated decrease in the alpha–delta ratio (yellow boxes) that indicates a sudden loss of fast activity. Finally, there is a decrease in the overall amplitude of integrated EEG (green boxes) across both hemispheres. EEG, electroencephalogram

A 69-year-old woman with acute myeloid leukemia presented for episodes of sudden impaired awareness and generalized shaking. She was initially diagnosed with seizures, but events persisted despite multiple antiseizure medications. Upon admission to the hospital, there were three events of interest recorded on continuous video electroencephalogram (EEG). Each event correlated on EEG with diffuse attenuation of fast activity, increased delta activity, and subsequent attenuation of all frequencies lasting 30–60 s (Fig. 1). Quantitative EEG detected a decrease in fast activity, alpha–delta ratio, and amplitude-integrated EEG (Fig. 2). The patient was not under any sedation during the events of interest.

We present the case of a 69-year-old woman with acute myeloid leukemia with recurrent transient events characterized by loss of awareness and generalized shaking. The events were initially diagnosed as seizures based on semiology but persisted despite anti-seizure medications. The events were recorded using continuous video electroencephalography (EEG) with no epileptiform correlate. However, pertinent changes included diffuse attenuation of fast activity, increase in delta activity, and subsequent attenuation of faster frequencies. Quantitative EEG detected a decrease in fast activity, alpha-delta ratio, and amplitude-integrated EEG. The transient events and EEG findings in the setting of known cancer history raised concern for carcinomatous meningitis despite unremarkable brain imaging. Lumbar puncture showed an elevated opening pressure and cytology confirmed a myeloid blast population consistent with acute myeloid leukemia. The events resolved with serial lumbar punctures supporting the fact that the events were likely secondary to transient elevations in intracerebral pressure. In summary, video EEG with quantitative EEG analysis is a sensitive, non-invasive way to confirm transient elevated intracranial pressure and rule out epileptic activity. This constellation of cancer, clinical symptoms and EEG findings should increase suspicion of carcinomatous meningitis.

Magnetic resonance imaging of the brain revealed no evidence of leptomeningeal disease. A lumbar puncture was pursued and revealed a moderately elevated opening pressure of 56 mm Hg. Cerebrospinal fluid analysis showed an elevated nucleated cell level (422 µL, reference 0–5 µL), an elevated protein level (68 mg/dL), and a normal glucose level. The meningitis panel and culture results were negative. Flow cytometry of the cerebrospinal fluid revealed a myeloid blast population consistent with acute myeloid leukemia. A bone marrow biopsy also confirmed acute myeloid leukemia.

The patient was treated with serial lumbar punctures along with intrathecal chemotherapy every three to seven days. She was also treated with systemic chemotherapy. No further events occurred after the initial lumbar puncture. The patient ultimately transitioned to hospice care in the setting of bacteremia, and more definitive intracerebral pressure treatment with a ventriculoperitoneal shunt was deferred. Although the elevated lumbar puncture opening pressure supports the diagnosis of elevated intracranial pressure (ICP), the absence of direct ICP monitoring with a device, such as a ventriculoperitoneal shunt, is a limitation.

In summary, video EEG with quantitative EEG analysis is a sensitive, noninvasive way to confirm transient elevated ICP and rule out epileptic activity. In the setting of malignancy, this constellation of clinical symptoms and EEG findings should increase suspicion of carcinomatous meningitis. This suspicion should remain present even though leptomeningeal disease may be absent in brain imaging.

## Declarations

## Conflict of interest

None of the authors have specific conflicts of interest related to this topic. E.G. has served as a site principal investigator for studies sponsored by Xenon, Eisai, and Stanford University. She serves on a Xenon advisory board. None of these relate to the current project.

## Ethical approval/informed consent

The authors confirm compliance with ethical approval and informed consent for human studies. The patient provided verbal and written consent for publication.

